# Single Versus Double Tourniquet Technique for Ultrasound-Guided Venous Catheter Placement

**DOI:** 10.5811/westjem.2019.7.43362

**Published:** 2019-08-06

**Authors:** Jacob Price, Jane Xiao, Katie Tausch, Bophal Hang, Amit Bahl

**Affiliations:** * St. Mary Mercy Hospital, Department of Emergency Medicine, Livonia, Michigan; † Oregon Health and Science University, Department of Emergency Medicine, Portland, Oregon; ‡ Oakland University William Beaumont School of Medicine, Department of Emergency Medicine, Royal Oak, Michigan

## Abstract

**Introduction:**

Peripheral, ultrasound-guided intravenous (IV) access occurs frequently in the emergency department, but certain populations present unique challenges for successfully completing this procedure. Prior research has demonstrated decreased compressibility under double tourniquet technique (DT) compared with single tourniquet (ST). We hypothesized that catheters inserted under DT method would have a higher first-stick success rate compared with those inserted under ST method.

**Methods:**

We randomized 100 patients with a history of difficult IV access, as defined by past ultrasound IV, prior emergency visit with two or more attempts required for vascular access, history of IV drug abuse, history of end stage renal disease on hemodialysis or obesity, to ultrasound-guided IV placement under either DT or ST method. We measured the vein characteristics measured under ultrasound, and recorded the number of attempts and location of attempts at vascular access.

**Results:**

Of an initial 100 patients enrolled, we analyzed a total of 99 with 48 placed under ST and 51 placed under DT. Attending physicians inserted 41.7% of ST and 41.2% of DT, with non-attending inserters (including residents, nurses, and technicians) inserted the remainder. First-stick success rate was observed at 64.3% in ST and 66.7% in DT (p=0.93). Attendings had an overall higher first-stick success rate (95.1%) compared to non-attending inserters (65.5%) (p=<0.001). The average vein depth measured in ST was 0.73 centimeters (cm) compared with 0.87 cm in DT (p=0.02).

**Conclusion:**

DT technique did not produce a measureable increase in first-stick success rate compared to ST, including after adjusting for level of training of inserter. However, a significant difference in average vein depth between the study arms may have limited the reliability of our overall results. Future studies controlling for this variable may be required to more accurately compare these two techniques.

## INTRODUCTION

Peripheral intravenous (IV) access is one of the most common invasive procedures performed in emergency departments (ED) and is frequently required for diagnosis and treatment of patients with a wide spectrum of conditions. A certain subset of patients including those with obesity, a history of IV drug abuse, chronic diseases, and acute hypovolemia presents a particular challenge in establishing IV access, which would otherwise be a routine intervention.^1^,^2^ Ultrasound-guided peripheral venous access (USGPIV) is an approach to establish IV access in patients with difficult vascular access (DVA),^3^ which can successfully be used by both emergency physicians (EP) and support staff.^8^–^10^

Past studies of USGPIV have reflected that larger vessels cannulate more easily.^11^ Here we specifically explore the effect of the double tourniquet (DT) method on successful IV cannulation. This method was previously demonstrated to decrease vein compressibility.^12^ While in the previous evaluation vein size and compressibility were measured, impact on successful insertion was not evaluated. The objective of this investigation was to test whether a DT placement enhances first-stick success with USGPIV cannulation compared to standard, single tourniquet (ST) placement.

## METHODS

### Study Design and Setting

This study was a prospective, randomized, comparative evaluation of single vs double tourniquet placement on first-stick, USGPIV cannulation success. It was approved by the institutional review board (IRB) at the home institution. We conducted the study in the ED of a suburban, academic teaching tertiary-care center with more than 130,000 visits per year. The IRB waived written informed consent for study participants and required that subjects be verbally consented. The sample size was based on enrollment feasibility and possibility of exploratory analyses; no prior power analysis was performed.

EPs (residents and attendings) and ED ancillary staff (nurses and technicians) who were proficient in USGPIV placement using single-user technique performed the US-guided peripheral IV insertion. We analyzed insertions under the grouping of attending physicians (42) vs a combination of resident physicians and ancillary staff (58). Departmental certification in US-guided vascular access involves attending a two-hour, vascular access didactic session followed by successful placement of US-guided IVs in the ED. The didactic session includes a discussion of relevant anatomy, insertion techniques, pitfalls, and training with the Blue Phantom 2 Vessel Ultrasound Training Block (CAE Healthcare, Sarasota, FL 34240). All inserters had at least one year of experience in this procedure. No specific training or refresher was offered prior to subject enrollment.

### Selection of Participants

Research staff and investigators recruited a convenience sample of DVA patients presenting to the ED between June–August 2018. Investigators consented patients and provided an information sheet outlining the study protocol. Study participation was voluntary, and consent was obtained prior to enrollment. Post consent, patients were randomized using an envelope system of randomization to either ST or DT group. This was simple randomization done by the biostatistics department using a computerized system with a 1:1 ratio for each group. The investigator opened the envelope once the eligibility was confirmed and the patient was consented.

Patients eligible for the study had to be at least 18 years of age, had to have failed a blind IV attempt in the current department visit, and been identified as a DVA patient with at least one of the following:

1. History of IV drug use.
2. End stage renal disease on hemodialysis.
3. History of needing a rescue catheter such as US-guided IV, central venous catheter, or peripherally inserted central catheter on a previous hospitalization.
4. At least two, blind, unsuccessful attempts during present visit
5. Patient request of an ultrasound-guided IV without prompting.

**Population Health Research Capsule**

What do we already know about this issue?

*Double tourniquet technique has demonstrated decreased vein compressibility as measured under ultrasound when performed in healthy volunteers*.

What was the research question?

*Does double tourniquet technique lead to improved ultrasound guided IV first stick success rate over single tourniquet*?

What was the major finding of the study?

*Double tourniquet technique did not demonstrate increased first stick success rate when compared to single tourniquet*.

How does this improve population health?

*Difficult IV access patients may benefit from ultrasound guided IV placement, but double tourniquet technique does not appear to improve success in placement*.

Patients were excluded if they voluntarily withdrew from the investigation.

### Assessment/Procedure

**Figure 1:**
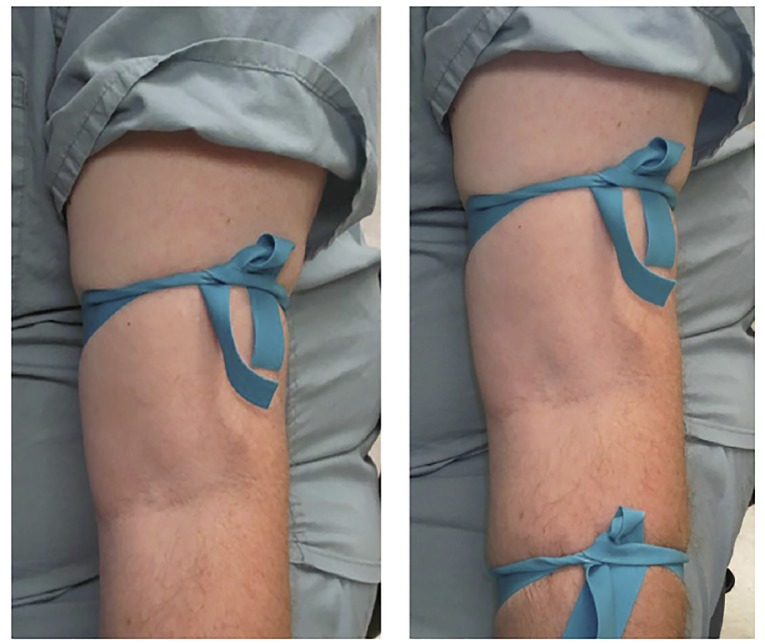
Standard tourniquet pictured left; double tourniquet pictured right.

Patients randomized to the ST had a single device placed on the arm or forearm at the discretion of the inserter, and those randomized to a DT had the initial tourniquet placed followed by a second approximately 30 centimeters (cm) distal to the first (Figure 1). In both cases, vein diameter was measured both prior to and after placement of a tourniquet.

The investigators were trained to perform uniform bedside assessment of the venous system including measuring and saving vessel depth and diameter. The linear array transducer was used for all insertions, either a L12-4s Mindray M9 unit (Mindray North America, San Jose, CA) or a HFL38xp Sonosite X-Porte (FUJIFILM Sonosite, Inc, Bothell, WA) depending on patient proximity to the device within the department. Investigators measured vessels in short-axis orientation and assessed diameter pre- and post- tourniquet. The US site director reviewed all scans and measurements for accuracy. There were no discrepancies in measurements of vein depth between the initial operators and the director’s review. A 4.78 cm 20-gauge, peripheral IV catheter was used for the evaluation. Investigators recorded first-stick success, number of attempts, and overall success of the procedure as well as the location of insertion (upper arm, antecubital, forearm) of all successful placements. A successful insertion was confirmed by full advancement of the catheter so that the catheter was no longer externally visible, immediate sampling of at least five cubic centimeters (cc) of blood, and flushing without resistance of five cc of saline.

Patient, IV, and vein characteristics at time of initial assessment were summarized for each study group using medians and interquartile ranges for continuous variables and compared by Kruskal-Wallis tests. We summarized frequencies and percentages for categorical variables and compared them by chi-squared tests or Fisher’s exact tests. Logistic regression was employed to assess the strength of association between type of IV inserters and first-stick success. All tests of statistical significance were two-sided with a p-value < 0.05 indicating a significant difference. We performed analyses using SAS version 9.4 (SAS Institute, Inc., Cary, NC).

Additional data collected from the electronic health record included age, gender, body mass index (BMI), vitals signs, and relevant past medical history.

The primary outcome was the first-stick success rate of obtaining venous access between the two groups. This was assessed by the number of attempts to establish access. We performed statistical comparisons using chi-squared and Fisher’s exact tests. All tests of statistical significance were two-sided with a p-value <0.05 indicating significant difference.

## RESULTS

**Figure 2:**
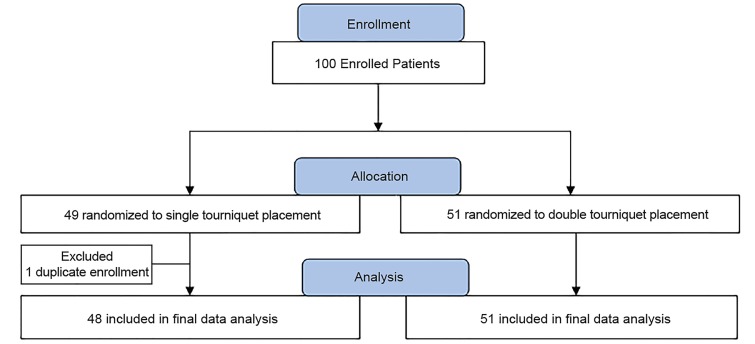
Enrollment Flowchart.

We consented and enrolled 100 patients in the study. Data from 99 patients was available for the final analysis. One patient was excluded due to prior enrollment discovered after randomization (Figure 2). Table 1 illustrates patient demographics with no statistical difference between groups regarding age, gender, BMI, and vital signs.

Veins were deeper in the DT group compared with the ST group at 0.73 cm vs 0.87 cm, respectively (p=0.02). After tourniquet application, veins dilated in both ST and DT groups. However, there was no significant difference in the amount of change in vein diameter between ST and DT groups. Vein characteristics prior to insertion are shown in Table 2.

First-stick success was similar in both groups with successful insertion in 38, or 79.2%, in the ST group vs 39, or 76.5%, in the DT group (p = 0.75). Overall success was similar in both groups with only one failure in each group resulting in 47 (97.9%) and 50 (98.0%) success in ST and DT groups respectively (p = 0.96). The two groups underwent similar rates of blind attempts prior to study enrollment with an average of 2.0 in each group (p = 0.82), and both groups yielded successful IV placement after an average of one attempt (p = 0.88). One failure in the DT group underwent five attempts at placement before opting out of further participation in the study.

Attending physicians and non-attending providers inserted catheters for this evaluation. Attendings inserted 20 in the ST group and 19 in the DT group, totaling 42% and 41% of total catheters inserted in each group respectively. Attending physicians were overall more successful with first attempt in 20 (100%) of ST cases and 19 (90.5%) of DT cases (p=0.49) compared to non-attendings’ first-stick success of 18 (64.3%) and 20 (66.7%) for ST and DT groups (0.85), respectively. Table 3 shows breakdown of first-stick success by type of inserter. Table 3 also shows significantly higher percentages of first-stick success of ST and DT placement by attending physicians (95%) compared to non-attending inserters (65%). Attending physicians are 10 times more likely to have first-stick successful IV placement in contrast to non-attendings (odds ratio: 10.3, 95% confidence interval, 2.2 to 46.9).

Further investigation regarding location of IV placement did demonstrate a lower success rate in the upper arm when compared to the antecubital and forearm; however, this difference did not approach clinical significance. This overall trend bore out when divided into ST and DT technique. Table 4 demonstrates vein location in the arm.

## DISCUSSION

Vein dilation devices have a role in USGPIV insertion as a larger venous target may facilitate IV insertion. The Esmarch bandage, Rhys-Davis exsanguinator, and a vacuum device have all been described as routes to augment vein size, in addition to the local application of nitroglycerine ointment.^13^–^15^ Tourniquets and blood pressure cuffs that may be applied for vein enhancement are readily available and ubiquitous in most EDs and inpatient wards. While blood pressure cuffs are pressure controlled and tightness around the arm can be uniformly standardized using this device, the blood pressure cuff also occupies a large portion of the arm, limiting location of sites for IV insertion to the antecubital fossa and forearm. Therefore, we chose to evaluate strategic application of a practical solution, namely tourniquets, for this intervention.

We used first-stick success as the primary outcome in our study as it is one of the quality and safety goals of our vascular access program. Past studies of difficult peripheral IV access have demonstrated that patients requiring multiple attempts or physician intervention could have a delay of access of up to two hours,^16^ which in turn can lead to a delay of both care and treatment. Repeated access attempts also correspond to more time spent by technician, nursing, and physician staff members away from other tasks. As equipment preparation has been identified as an area of increased risk for needlestick injuries,^17^ attempts beyond the first may represent a hazard to the inserter as well.

Unquestionably, multiple punctures by a needle and increased tourniquet time are uncomfortable for patients from both a physical and at times psychological perspective.^18^ Additionally, repeated venipuncture performed prior to successful line placement may result in increased vessel wall damage, which in turn raises concern for development of phlebitis and even the potential for thrombus formation. Ultrasound IVs have also been identified with increased risk of extravasation upon administration of contrast for computed tomography.^19^ Damage to the endothelium from multiple attempts may raise this risk as well.

Attending faculty experienced a very high first-stick success with 100% in the ST cohort and 90.5% in the DT cohort. As inserters become more experienced with the procedure, the potential benefit of adding a tourniquet also diminishes and would require a very large sample size to demonstrate whether a statistical difference truly existed. Resident, nurse, and technician inserters had very similar rates of success between arms, 64.3% in ST vs 66.7% in DT, and while a larger study may reveal an actual difference in success rates, our results do not suggest significant benefit to an added tourniquet.

While location of the vessel did not favor one location to the point of clinical significance, a trend toward increased success in the forearm and antecubital location was noted. While past studies have focused more upon longevity than ease of cannulation in comparison of access sites,^20^ further investigation to compare the success rates between locations may be warranted. If the upper arm truly is more difficult to access upon initial puncture in addition to having less robust longevity, pursuing it as a site of access may be more appropriate as a back-up choice.

## LIMITATIONS

There were several limitations to our investigation. We did not account for compressibility of the vein in this trial, the key difference observed between double and single tourniquet in previous comparisons of the techniques.^12^ Although we measured vein dilation in both groups, it is possible that compressibility may impact successful cannulation independent of vein size. As standard tourniquets do not have pressure control capability, differences in vein dilation and compressibility may partly be due to the degree of tightness with which the tourniquet was applied.

In a previous comparison of ST vs DT, tourniquet placement was standardized and specific vessels in the arm were measured.^12^ In our study, the selection of vessel and subsequent tourniquet placement was left to the discretion of the inserters in anticipation of variation of vein depth and accessibility across our patient population. We also believe this more realistically reflects the clinical process of establishing US-guided IV access with variation in inserter preference or comfort regarding vessel selection. Standardization of device placement and vein in question may have demonstrated a more consistent effect within each and between the two groups as well as providing a more consistent vein depth across the study.

There was a statistically significant difference in depth of vein between groups, and this may have impacted first-stick success rates. Vein depth is related to success of US-guided IV cannulation. Although the literature supports a depth of 1.20 cm as being a distance with increased failures of insertion,^20^ moving from 0.73 cm to 0.87 cm can create more difficulty with insertion and threading of the catheter. Deeper vessels can also run into challenges with seating a sufficient length of catheter in the vessel. A decreased percentage of the catheter residing in the vessel has been strongly associated with a higher hazard of failure,^21^ although this appears to reflect more directly on the catheter’s longevity rather than the ease of placement. However, a deeper vein may also require insertion at a steeper angle to achieve a greater length in the vessel, and steeper angles in turn can create challenges with successfully advancing a catheter without vessel injury or backwalling.

Finally, we performed no power analysis in our study. Our goal was simply to recruit a convenience sample of subjects over a limited amount of time; therefore, we did not conduct a sample size calculation or power analysis. Due to the lack of published data on this particular technique, key assumptions required for a calculation of sample size were not available. Nonetheless, our study may have been underpowered to reveal a significant difference in success between the two techniques.

## CONCLUSION

Single tourniquet vs double tourniquet technique does not impact first-stick success of the provider inserting the IV, regardless of his or her level of experience. Further investigations comparing these techniques under standardized technique and depth are needed to fully assess whether an added tourniquet provides any added success in first-stick success.

## Figures and Tables

**Table 1:** Patient Characteristics.§

	ST (n=48)	DT (n=51)	p-value
Age	58 (43, 75)	62 (43, 75)	0.64
Female (%)	34 (72.3%)	42 (82.3%)	0.23
History difficult access (%)	31 (64.6%)	32 (62.7%)	0.85
IV drug use (%)	3 (6.2%)	3 (5.9%)	1.00
Hemodialysis (%)	10 (20.8%)	7 (13.7%)	0.35
Obese (%)	24 (50.0%)	25 (58.9%)	0.49
Body Mass Index	30.1 (25.8, 36.0)	30.2 (25.1, 37.1)	0.72
Vital signs			
Systolic BP	137.5 (117.0, 154.0)	131.0 (118.0, 151.0)	0.73
Diastolic BP	71.5 (58.0, 89.0)	74.0 (58.0, 89.0)	0.94
HR	88.5 (74.5, 108.0)	90.0 (78.0, 101.0)	0.84
RR	18.0 (18.0, 20.0)	18.0 (18.0, 22.0)	0.24
Temperature, °C	36.9 (36.6, 37.0)	36.8 (36.6, 37.0)	0.90

*ST*, single tourniquet; *DT*, double tourniquet; *BP*, blood pressure; *HR*, heart rate; *RR*, respiratory rate.

§ For continuous variables, medians (interquartile ranges) were presented.

For categorical variables, frequencies (percentages) were presented.

**Table 2:** Vein Characteristics.§

	ST (n=48)	DT (n=51)	p-value
Depth of vein, cm	0.73 (0.61, 0.94)	0.87 (0.71, 1.27)	0.02
Pre-diameter of vein, cm	0.25 (0.20, 0.32)	0.24 (0.20, 0.32)	0.86
Post-diameter of vein, cm	0.29 (0.23, 0.37)	0.29 (0.22, 0.37)	0.73
Change of vein diameter, cm	0.04 (0.01, 0.06)	0.03 (0.01, 0.08)	0.59

*ST*, single tourniquet; *DT*, double tourniquet; *cm*, centimeter.

§ For continuous variables, medians (interquartile ranges) were presented.

For categorical variables, frequencies (percentages) were presented. There were 9 to 12 missing observations on depth of vein and diameter of vein in each group.

**Table 3:** IV Characteristics at time of initial assessment.§

	ST	DT	p-value
n	48	51	
IV placed by (%)			
Attending physicians	20 (41.7%)	21 (41.2%)	0.96
Non-attending inserters	28 (58.3%)	30 (58.8%)	
IV location in arm (%)			
Antecubital	21 (44.7%)	30 (60.0%)	0.27
Forearm	14 (29.8 %)	9 (18.0%)	
Upper	12 (25.5%)	11 (22.0%)	
First-stick success (%)			
Yes	38 (79.2%)	39 (76.5%)	0.75
No	10 (20.8%)	12 (23.5%)	
Overall success (%)			
Yes	47 (97.9%)	50 (98.0%)	0.96
No	1 (2.1%)	1 (2.0%)	
Number of blind attempts	2.0 (1.0, 3.0)	2.0 (1.0, 3.0)	
Number of attempts	1.0 (1.0, 1.0)	1.0 (1.0, 1.0)	
IV placed by attending physicians (n=41)			
n	20	21	
First-stick success (%)			
Yes	20 (100.0%)	19 (90.5%)	0.49
No	0 (0.0%)	2 (9.5%)	
IV placed by non-attending inserters (n=58)			
n	28	30	
First-stick success (%)			
Yes	18 (64.3%)	20 (66.7%)	0.85
No	10 (35.7%)	10 (33.3%)	

*ST*, single tourniquet; *DT*, double tourniquet.

§ For continuous variables, medians (interquartile ranges) were presented. For categorical variables, frequencies (percentages) were presented.

There was a missing observation on IV location in each group.

**Table 4:** Results of intravenous placement by type of location in arm.§

	Single tourniquet		p-value
IV location in arm (%)	Success (n=38)	Failure (n=9)	0.41
Antecubital	18 (85.7%)	3 (14.3%)	
Forearm	12 (85.7%)	2 (14.3%)	
Upper	8 (66.7%)	4 (33.3%)	

	Double tourniquet		p-value

IV location in arm (%)	Success (n=39)	Failure (n=11)	0.40
Antecubital	24 (80.0%)	6 (20.0%)	
Forearm	8 (88.9%)	1 (11.1%)	
Upper	7 (63.6%)	4 (36.4%)	

	All (single/double tourniquet)		p-value

IV location in arm (%)	Success (n=77)	Failure (n=20)	0.14
Antecubital	42 (82.3%)	9 (17.7%)	
Forearm	20 (87.0%)	3 (13.0%)	
Upper	15 (65.2%)	8 (34.8%)	

§ There was a missing observation on IV location in each group.
